# Association of host immunity with *Helicobacter pylori* infection in recurrent gastric cancer

**DOI:** 10.1186/s13027-019-0221-1

**Published:** 2019-02-11

**Authors:** Mayu Sato, Kou Miura, Chihiro Kageyama, Hiroyuki Sakae, Yuka Obayashi, Yoshiro Kawahara, Osamu Matsushita, Kenji Yokota, Hiroyuki Okada

**Affiliations:** 1 Graduate School of Health Science, Okayama University, 2-5-1 Shikata-cho Kita-ku, Okayama, 700-8558 Japan; 2 Gastroenterology and Hepatology, Medicine, Dentistry and Pharmaceutical Sciences, Okayama University, Okayama, Japan; 3 Bacteriology, Medicine, Dentistry and Pharmaceutical Sciences, Okayama University, Okayama, Japan

**Keywords:** *Helicobacter pylori*, IL-10, Single nucleotide polymorphism, IgG subclass

## Abstract

**Background:**

*Helicobacter pylori* infection is associated with the incidence of gastric cancer. Endoscopic resection has been developed as a proper technique to treat early stage of gastric cancer. However, some patients develop recurrent gastric cancer within 5 years after endoscopic treatment. The aim of the present study is to explore a biomarker for detecting people who has high risk of gastric cancer recurrence.

**Methods:**

We analyzed the Interleukin-10 (IL-10) single nucleotide polymorphism (SNP) and IgG subclass responses to the bacteria in patients with early gastric cancer and recurrent gastric cancer.

**Results:**

Patients with hetero-type in the 1082 SNP and CC genotype in the 592 SNP were at high risk of recurrence of gastric cancer. In patients with genotype carrying high risk of recurrence, IgG1 level tended to be higher than that in patients with other genotypes.

**Conclusions:**

Dominance of T helper 2 (Th2) immunity controlled by IL-10 cytokine may be associated with *H. pylori*-associated gastric cancer recurrence.

## Background

*Helicobacter pylori* infection is an important factor associated with gastric cancer [1]. Incidence of *H. pylori* induced gastric cancer is dependent on geographic factors, strain diversity, and host immunological responses [2]. The incidence of gastric cancer is higher in East Asian countries than other parts of the world [3, 4]. Therefore, several of the strain-specific features linked to high gastric cancer risk (including the cagA-ABD type of PAI, type s1 forms of vacA and babA) are present in nearly all East Asian *H. pylori* isolates [5–7]. However, the only minority patients were finally developed gastric cancer in the high carcinogenic *H. pylori* infected many patients. It is difficult to easily find a patient who develops gastric cancer among infected people.

Recently, endoscopic examination and resection has been established as a proper technique to treat early gastric cancer [8]. However, some patients developed recurrent gastric cancer within 5 years after endoscopic treatment. Previous studies reported that the incidence of recurrent gastric cancer within 5 years after endoscopic treatment is 3–10% [9, 10]. Clinicians recommend endoscopic examination every year, as it is difficult to distinguish patients with recurrent cancer. Therefore, identification of patients with high risk of recurrent cancer will be clinically and economically beneficial to the patients. We thought host immune response might be a potential marker to identify the patients with high risk of recurrent cancer.

*H. pylori* infection usually induces a strong T helper 1 (Th1) inflammatory response, which is characterized by cellular infiltration induced by cytokine and chemokine secretion. During the pathogenesis from chronic gastritis to gastric cancer caused by *H. pylori* infection, activated neutrophils and mononuclear cells in the host can produce pro-inflammatory cytokines, such as interleukin IL-1β, IL-6, IL-8, and tumor necrosis factor (TNF)-α, and anti-inflammatory cytokines such as IL-10 [11, 12]. Therefore, host immunity is closely associated with gastric cancer risk, since gastric cancer usually develops in patients after more than 30 years of *H. pylori* infection.

In the context of anti-*H. pylori* immunity, studies on serum anti*-H. pylori* IgG subclass have indicated that subjects with low levels of IgG2 anti-*H. pylori* antibody were at a risk of gastric cancer [13, 14], suggesting that a decreasing Th1 response is associated with developing gastric cancer. IL-10 inhibits the production of pro-inflammatory cytokines by inhibition of Th1 lymphocytes, and stimulation of B lymphocytes and Th2 lymphocytes and thus downregulates the inflammatory response [15–17].

There are three frequently detected SNPs of IL-10 promoter gene, at position-1082 A/G and − 819 C/T SNPs in its proximal promoter region and at position − 592 A/C SNP in its 5′-flanking region, which are associate with IL-10 production and development of gastric cancer [18–20]. The IL-10 promoter was also reported to be affected by the production of IL-10 [19–21].

In this study, we analyzed the IL-10 SNPs and IgG subclass responses to the bacteria in patients with early gastric cancer and recurrent gastric cancer.

## Materials and methods

### Patient information and DNA extraction

Samples from the patients were collected in Okayama University hospital with patients’ informed consent. Patients were treated by endoscopic mucosal resection (EMR) and followed up from 1 to 5 years. Patients’ information was shown in Table 1, and clinical character of recurrent gastric cancer patients was shown in Table 2. We obtained 10 to 20 paraffin sections of stomach tissue after first EMR of 49 cases of gastric cancer (20 cases of recurrence, 29 cases of recurrence within 5 years). DNA was extracted from these paraffin sections using PAX gene® Tissue DNA Kit and was stored at − 80 °C.

**Table 1:** Patient characteristics

	Recurrent	Not recurrent	*P* value
	*n* = 20	*n* = 29	
Mean age, year (±SD)	72.1 (7.9)	65.2 (7.6)	0.002
Gender (F/M)	4/16	8/21	n. s.
Observation period (month) median (range)	30(33–118)	82(26–99)	0.03
Endoscopic atrophy (open/closed)	19/3	20/9	n. s

**Table 2:** Clinical character of recurrent gastric cancer patients

	Initial cancer			Recurrent cancer		
locate	L	M	U	L	M	U
	6	12	3	6	8	6
Macroscopic classification	IIa	IIb	IIc	IIa	IIb	IIc
	9	0	10	8	1	12
tissue type	tub1	tub2	other	tub1	tub2	other
	14	7	0	15	3	2

### PCR

The following primers were designed to amplify the IL-10 promoter region containing three SNPs for PCR: IL-10 forward primer (IL-10F) 5′-GTG GAA GGG GAA GGT GAA-3′ and IL-10 reverse primer (IL-10R) 5′-CCC AAG ACT TCT CCT TGC TA-3′. The cycling conditions consisted of an initial single cycle of a min at 98 °C, followed by 35 cycles of 10s at 98 °C, 30s at 60 °C, and 60s at 72 °C. Subsequently, the PCR product was diluted 50 times and a second PCR was performed using SNP-detection primers designed for the IL-10 region (Table 3). The cycling conditions when using primers of 819F, 819R, and 1082A-SNP consisted of an initial single cycle of a min at 98 °C, followed by 35 cycles of 10 s at 98 °C, 30 s at 66.5 °C, and 60 s at 72 °C. The annealing temperature was changed for the SNP detection primer without changing the other conditions; it was 66.5 °C for 1082G-SNP (same as 1082A-SNP), 57 °C for 819 T-SNP and 592A-SNP, 63 °C for 819C-SNP, and 60 °C for 592C-SNP. The obtained PCR product was electrophoresed on a 1% agarose gel at 100 V for 30 min, stained with ethidium bromide for 10 min, and then photographed.

**Table 3:** Primer information

Primer	Sequences of primers	Melting Temperature (°C)	Annealing Temperature (°C)
IL-10F	5′-GTG GAA GGG GAA GGT GAA-3’	55.8	60.0
IL-10R	5’-CCC AAG ACT TCT CCT TGC TA-3’	56.3	
819F	5’-GAC TCC AGC CAC AGA AGC TTA C-3’	60.4	^a^
819R	5′-AGG TCT CTG GGC CTT AGT-3′	55.8	
1082A-SNP	5’-AAC ACT ACT AAG GCT TCT TTG TGA-3’	57.3	66.5
1082G-SNP	5’-AAC ACT ACT AAG GCT TCT TTG TGA GG-3’	58.9	66.5
819 T-SNP	5’-TAC CCT TGT ACA GGT GAT GGA ATA-3’	57.1	57.0
819C-SNP	5’-TAC CCT TGT ACA GGT GAT GGA ACA-3’	58.8	63.0
592A-SNP	5′-TGA CCC CGC CGG TAC-3’	52.0	57.0
592C-SNP	5′- TGA CCC CGC CGG TCC-3’	54.0	60.0

^a^Depend on the SNP detection primer

### ELISA

The levels of serum IgG subclass and total IgG antibodies against *H. pylori* antigen (*cagA*; ABD, *vacA*; s1c/m1, *cagE*+, *iceA*1, and *babA*2) were measured by ELISA. Ninety-six-well microtiter plates were coated with *H. pylori* lysate (50 μg/ml) in 100 μl of 0.1 M carbonate-bicarbonate buffer (pH 9.6) and incubated overnight at 4 °C. After the wells were blocked with Phosphate Buffered Saline (PBS) containing 10% skim milk, the plates were incubated with sera containing 1:1000 of IgG and 1:100 of IgG1/2 for 2 h and washed with PBS containing 0.05% Tween 20. Peroxidase-labeled rabbit anti-human IgG1, IgG2, or IgG antibody (Dako Japan) was then added subsequently, and the plates were incubated for 2 h. After the plates were washed, 1 mg/ml of o-phenylenediamine (Wako Pure Chemical) in citrate buffer (pH 5.5) was added to the wells. The ODs were measured at 490 nm using an ELISA plate reader (Bio-Rad).

### Statistical analysis

The recurrence/non-recurrence rate in each genotype was statistically analyzed using Fisher’s exact probability test. Welch’s t-test was used for statistical analysis of each value of the IgG subclass. In both cases, *p* < 0.05 was considered significant, and a statistical software program (4 Steps Excel Statistics Ver.2, OMS, Japan) was used for all calculations.

## Results

### IL-10 polymorphisms with gastric cancer recurrence rate

**Fig. 1:**
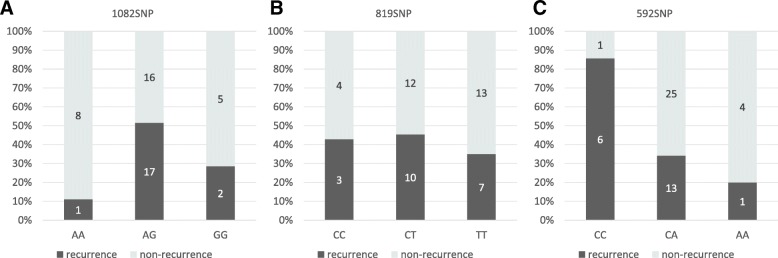
IL-10 polymorphisms with gastric cancer recurrence rate. Association with **a**: 1082SNP(AA, AG, and GG), **b**: 819SNP(CC, CT, and TT), and **c**: 592SNP(CC, CA, and AA) and each recurrence rate are shown

The recurrence/non-recurrence rate of gastric cancer in the genotype for each SNP is shown in Fig. 1. The 1082 SNP was classified into homo genotype (AA, GG) and hetero genotype (AG); incidence of recurrent gastric cancer decreased in homo genotype (10% of AA and 28% of GG) of the 1082 SNP (Fig. 1a). A statistically significant difference was found between AA and AG genotypes in the 1082 SNP (*p* < 0.05). Eight hundred and-nineteen SNP was not associated with recurrent gastric cancer (Fig. 1b). Further, in the 592 SNP, a significantly (*p* < 0.05) high rate (86%) of CC genotype was associated with recurrent gastric cancer (Fig. 1c).

### Association with allele/haplotype frequencies and recurrent/non-recurrent

Frequencies of ATA haplotype (1082A, 819 T, 592A) in non-recurrent gastric cancer were significantly higher than those in recurrent gastric cancer (Table 4). However, the GCC haplotype (1082G, 819C, 592C) was significantly increased in recurrent gastric cancer.

**Table 4:** Allele/haplotype frequencies of gastric cancer recurrence

	Frequencies (%)*	
	ATA+	GCC+
Recurrent (*n* = 20)	11 (55%)	12 (60%)
Non-recurrent (*n* = 29)	22 (79%)	11 (38%)

*p = 0.0031 by chi-square test

### IgG antibody level in recurrent gastric cancer

**Fig. 2:**
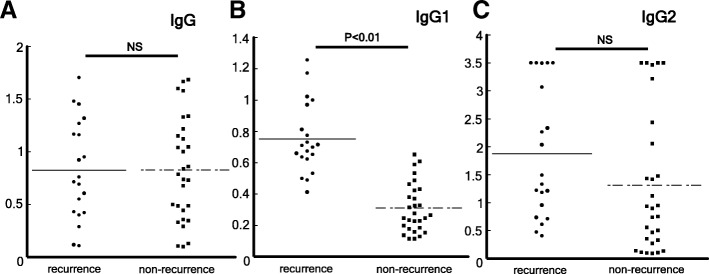
IgG antibody in patients with recurrent or not-recurrent gastric cancer. IgG and IgG2 antibody levels in patients with recurrent or not-recurrent were not different. IgG1 antibody level in patients with recurrent gastric cancer was significantly higher than not-recurrent patients

**Fig. 3:**
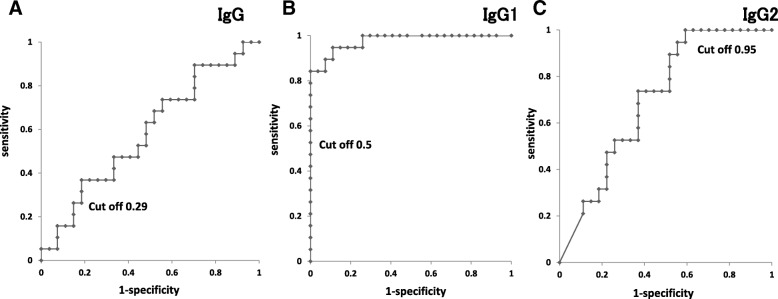
The ROC curves of IgG and IgG subclass in recurrent or non-recurrent gastric cancer. Total IgG (**a**), IgG1 (**b**), and IgG2 (**c**) to *H. pylori* in patients with recurrent or non-recurrent are shown. Cut off values of IgG, IgG1, and IgG2 are 0.29. 0.5, and 0.95, respectively

The levels of total IgG and IgG subclass (IgG1 and IgG2) against *H. pylori* were measured (Fig. 2). IgG1 antibody was significantly higher in patients with recurrent cancer. To evaluate the diagnostic performance, ROC analysis was used. The ROC curves of IgG and IgG subclass are shown in Fig. 3. AUC values of total IgG, IgG1 and IgG2 were 0.58, 0.98, and 0.70, respectively.

## Discussion

Gastric cancer remains one of the most common causes of cancer-related death. The incidence of gastric cancer prevails in a few cases among patients with *H. pylori* infection. Endoscopic resection has been established as a proper technique to treat early gastric cancer [19]. Some patients developed recurrent gastric cancer within 5 years after endoscopic treatment. Therefore, it is important to identify patients with high risk of recurrent gastric cancer. Some reports have indicated that IL-10 response and T helper polarity were associated with gastric cancer development. IL-10 strongly inhibits Th1 immunity, and Th1/Th2 balance affects producing IgG1/IgG2 balance. On the other hand, three SNPs of IL-10 promoter were effective to IL-10 production [21]. However, association with the SNPs and IgG1/IgG2 balance is obscure. No clear relationship was found between SNPs and IL-10 in this human study. Our study indicated that these host immunological feature and analysis of SNPs in the IL-10 promoter can be used to follow up after endoscopic resection of early gastric cancer. IgG1 (Th2 dominant antibody) response were available for prediction of recurrent gastric cancer.

## Conclusion

The 1082 and 592 SNPs may be associated with gastric cancer recurrence. Patients with hetero-type in the 1082 SNP and CC genotype in the 592 SNP possess high risk of recurrence of gastric cancer. In these patients, systemic IgG1 level tended to be higher than other genotypes. Frequencies of ATA, GCC haplotype were associated with recurrent gastric cancer. Therefore, Th1/Th2 immunity balance controlled by IL-10 may be associated with the risk of *H. pylori*-associated gastric cancer recurrence.

## Abbreviations

DNA: Deoxyribonucleic acid
ELISA: Enzyme-Linked ImmunoSorbent Assay
EMR: Endoscopic mucosal resection
Ig: Immunoglobulin
IL: Interleukin
PAI: Plasminogen activator inhibitor
PBS: Phosphate Buffered Saline
PCR: Polymerase chain reaction
SNP: Single nucleotide polymorphism
Th: T helper
TNF: Tumor necrosis factor
